# Histologic subtype-based evaluation of recurrence and survival outcomes in patients with adenocarcinoma of the ampulla of Vater

**DOI:** 10.1038/s41598-023-42386-6

**Published:** 2023-10-02

**Authors:** Se Jun Park, Kabsoo Shin, Tae Ho Hong, Sung Hak Lee, In-Ho Kim, Younghoon Kim, MyungAh Lee

**Affiliations:** 1 Division of Medical Oncology, Department of Internal Medicine, Seoul St. Mary’s Hospital, College of Medicine, The Catholic University of Korea, 222 Banpo-daero, Secho-gu, Seoul, Korea; 2 Cancer Research Institute, College of Medicine, The Catholic University of Korea, 222 Banpo-daero, Secho-gu, Seoul, Korea; 3 Department of General Surgery, Seoul St. Mary’s Hospital, College of Medicine, The Catholic University of Korea, Seoul, Korea; 4 Department of Pathology, Seoul St. Mary’s Hospital, College of Medicine, The Catholic University of Korea, 222 Banpo-daero, Secho-gu, Seoul, Korea

**Keywords:** Cancer, Oncology

## Abstract

Patients with ampulla of Vater adenocarcinoma exhibit diverse outcomes, likely since these malignancies can originate from any of the three converging epithelia at this site. Such variability presents difficulties in clinical decision-making processes and in devising therapeutic approaches. In this study, the potential clinical value of histomolecular phenotypes was determined by integrating histopathological analysis with protein expression (MUC1, CDX2, CK20, and MUC2), in a cohort of 87 patients diagnosed with stage IB to III ampulla of Vater adenocarcinoma who underwent curative surgical resection. Of the 87 patients, 54 were classified as pancreato-biliary (PB) subtype and 33 as intestinal subtype. The median follow-up time for all patients was 32.8 months (95% CI, 25.3–49.2). Patients with a histomolecular PB phenotype (CDX2 negative, MUC1 positive, MUC2 negative, and irrespective of the CK20 results) were associated with poor prognostic outcomes in both disease-free survival (DFS) (HR = 1.81; 95% CI, 1.04–3.17; *p* = 0.054) and overall survival (OS) (HR = 2.01; 95% CI, 1.11–3.66; *p* = 0.039) compared to those with histomolecular intestinal carcinomas. Patients with the PB subtype were more likely to have local recurrence alone (11 of 37, 29.7%) compared to those with the intestinal subtype (1 of 15, 6.7%). In the context of systemic disease, a notably greater proportion of patients exhibiting elevated carbohydrate antigen 19–9 levels were observed in the PB subtype compared to the intestinal subtype (*p* = 0.024). In the cohort of 38 patients who received first-line palliative chemotherapy, a diminished median overall survival (OS) was observed in the PB group compared to the intestinal group (10.3 vs. 28.3 months, HR = 2.47; 95% CI, 1.23–4.95; *p* = 0.025). By integrating histopathologic and molecular criteria, we can identify distinct and clinically relevant histomolecular phenotypes in adenocarcinomas of the ampulla of Vater, which could have considerable impact on existing therapeutic approaches.

## Introduction

The ampulla of Vater (AoV) represents a small anatomical area consisting of the junction between the pancreatic duct and the distal common bile duct, leading to the second portion of duodenum. The ampullary papilla is formed by intestinal epithelium, while the surrounding regions are covered by mucinous epithelium derived from the pancreato-biliary (PB) duct^1^. Due to these distinctions, AoV carcinoma can emerge from two diverse origins, leading to varied clinical outcomes. Consequently, a multitude of previous studies have examined the prognostic implications of histological and molecular subtypes in AoV cancer^2,3^.

Kimura et al*.* initially proposed a subclassification of AoV carcinoma into intestinal and PB phenotypes based on histologic characteristics, with the intestinal phenotype being associated with better survival outcomes^4^. Numerous subsequent studies have sought to refine this histologic classification using histological features and immunohistochemical markers^2,5–7^. The intestinal phenotype of AoV carcinoma, characterized by tubular or cribriform glands similar to those in colon cancer, exhibits expression of CDX2, MUC2, and CK20. In contrast, the PB phenotype, which resembles pancreatic ductal adenocarcinoma or extrahepatic bile duct cancer, typically shows positive staining for MUC1, mucin 5A, and cytokeratin 7^8^. Ang et al*.* introduced an approach utilizing a panel of four immunohistochemical markers, including MUC1, CDX2, CK20, and MUC2, to categorize mixed subtypes as either intestinal or PB phenotypes^2^.

Several previous studies have demonstrated that AoV carcinoma with a PB phenotype exhibits worse survival outcomes compared to those with an intestinal subtype. Chang et al*.* subclassified AoV carcinoma based on CDX2 and MUC1 expression, finding that the PB phenotype (MUC1 positive and CDX2 negative) correlated with a poorer prognosis^3^. Another study employing the same classification method confirmed that the PB phenotype was associated with worse survival outcomes in a multivariate analysis that included other risk factors^9^. Furthermore, the PB phenotype of AoV carcinoma was observed to have a higher tumor stage and increased risk of lymph node involvement^10^. However, the limited patient numbers and inconsistent subtype definitions in most studies make it challenging to evaluate the prognostic significance of histologic classification. Furthermore, assessment of patterns of recurrence and efficacy of chemotherapy based on histological subtype has not been fully explored.

In this study, the protein expression of MUC1, CDX2, CK20 and MUC2 was evaluated using immunohistochemistry (IHC) labeling on tissue microarray (TMA) samples from surgically resected AoV carcinoma. Subtypes were identified based on histological morphology and IHC results and were analyzed for recurrence patterns and survival outcomes.

## Results

### Patients characteristics

From August 1, 2007, through December 31, 2021, a total of 87 patients were qualified for inclusion in this study. Of these, 54 cases (62.1%) were classified as the PB subtype, and the remaining 33 cases (37.9%) were categorized as the intestinal subtype. Table 1 presents the clinicopathological characteristics of the patients. The primary tumor stage appeared to be more advanced in the PB subtype than in the intestinal subtype, although not reaching statistical significance (T3-4, 53.7% vs. 33.3%; *p* = 0.064). The distribution of other clinicopathological parameters was comparable between the two groups. Additionally, no significant disparities were noted in the preoperative levels of tumor markers, carbohydrate antigen 19–9 (CA 19–9), and carcinoembryonic antigen (CEA), when assessed across histologic subtypes.

**Table 1:** Clinicopathological characteristics of patients with surgically treated ampulla of Vater carcinoma, stratified by histologic subtypes.

Variables	Total (*n* = 87)	PB type(*n* = 54)	Intestinal type(*n* = 33)	χ^2^ or *t*	df	*p* value
Age, Median (Range)	65 (39–87)	66 (39–87)	65 (42–87)	0.265	65	0.792
Gender, n (%)						
Male	49 (56.3)	29 (53.7)	20 (60.6)	0.397	1	0.529
Female	38 (43.7)	25 (46.3)	13 (39.4)			
Tumor size, cm, mean ± SD	2.5 ± 1.1	2.4 ± 1.0	2.5 ± 1.1	0.39	63	0.698
Histologic grading, n (%)						
Grade 1	15 (17.2)	7 (13.0)	8 (24.2)	1.826	1	0.177
Grade 2/3	72 (82.8)	47 (87.0)	25 (75.8)			
Resection margin, n (%)						
R0	86 (98.9)	53 (98.1)	33 (100)			1
R1	1 (1.1)	1 (1.9)	0			
Tumor Stage, n (%)						
T1–2	47 (54.0)	25 (46.3)	22 (66.7)	3.422	1	0.064
T3–4	40 (46.0)	29 (53.7)	11 (33.3)			
Node Stage, n (%)						
N0	37 (42.5)	21 (38.9)	16 (48.5)	0.772	1	0.38
N1–2	50 (57.5)	33 (61.1)	17 (51.5)			
TNM Stage, n (%)						
Stage I–II	38 (43.7)	22 (40.7)	16 (48.5)	0.499	1	0.48
Stage III–IV	49 (56.3)	32 (59.3)	17 (51.5)			
Lymphatic invasion, n (%)						
No	37 (42.5)	22 (40.7)	15 (45.5)	0.186	1	0.666
Yes	50 (57.5)	32 (59.3)	18 (54.5)			
Vascular invasion, n (%)						
No	68 (78.2)	42 (77.8)	26 (78.8)	0.012	1	0.912
Yes	19 (21.8)	12 (22.2)	7 (21.2)			
Perineural invasion, n (%)						
No	64 (73.6)	39 (72.2)	25 (75.8)	0.132	1	0.717
Yes	23 (26.4)	15 (27.8)	8 (24.2)			
Adjuvant chemotherapy, n (%)						
No	44 (50.6)	26 (48.1)	18 (54.5)	0.335	1	0.563
Yes	43 (49.4)	28 (51.9)	15 (45.5)			
Preoperative CA19-9 level, n (%)						
Within normal (< 40U/mL)	39 (44.8)	20 (37.0)	19 (57.6)	3.774	1	0.151
Above normal (≥ 40U/mL)	40 (46.0)	29 (53.7)	11 (33.3)			
Missing data	8 (9.2)	5 (9.3)	3 (9.1)			
Preoperative CEA level, n (%)						
Within normal (< 3.8 ng/mL)	59 (67.8)	36 (66.7)	23 (69.7)			0.943
Above normal (≥ 3.8 ng/mL)	9 (10.3)	6 (11.1)	3 (9.0)			
Missing data	19 (21.9)	12 (22.2)	7 (21.3)			

*PB*, pancreato-biliary; *df*, degrees of freedom; *SD*, standard deviation; *TNM*, tumor, node, metastasis; CA 19–9, carbohydrate antigen 19–9; *CEA*, carcinoembryonic antigen.

### Survival outcomes

The median follow-up time for all patients was 32.75 months (95% CI, 25.34–49.15). Recurrence and death occurred in 52 patients (59.8%) and 45 patients (51.7%), respectively. The median disease-free survival (DFS) for the entire cohort was 17.2 months (95% CI, 5.07–29.3), and the 1-year and 2-year DFS rates were 64.2% (95% CI, 52.9%–73.5%) and 45.7% (95% CI, 34.5%–56.2%), respectively. The median overall survival (OS) for the entire cohort was 55.2 months (95% CI, 38.4–71.9), and the estimated OS rates were 75.6% (95% CI, 64.7%–83.5%) at 2 years and 46.6% (95% CI, 34.2%–58.0%) at 5 years.

### Histologic subtypes and survival analyses

**Figure 1:**
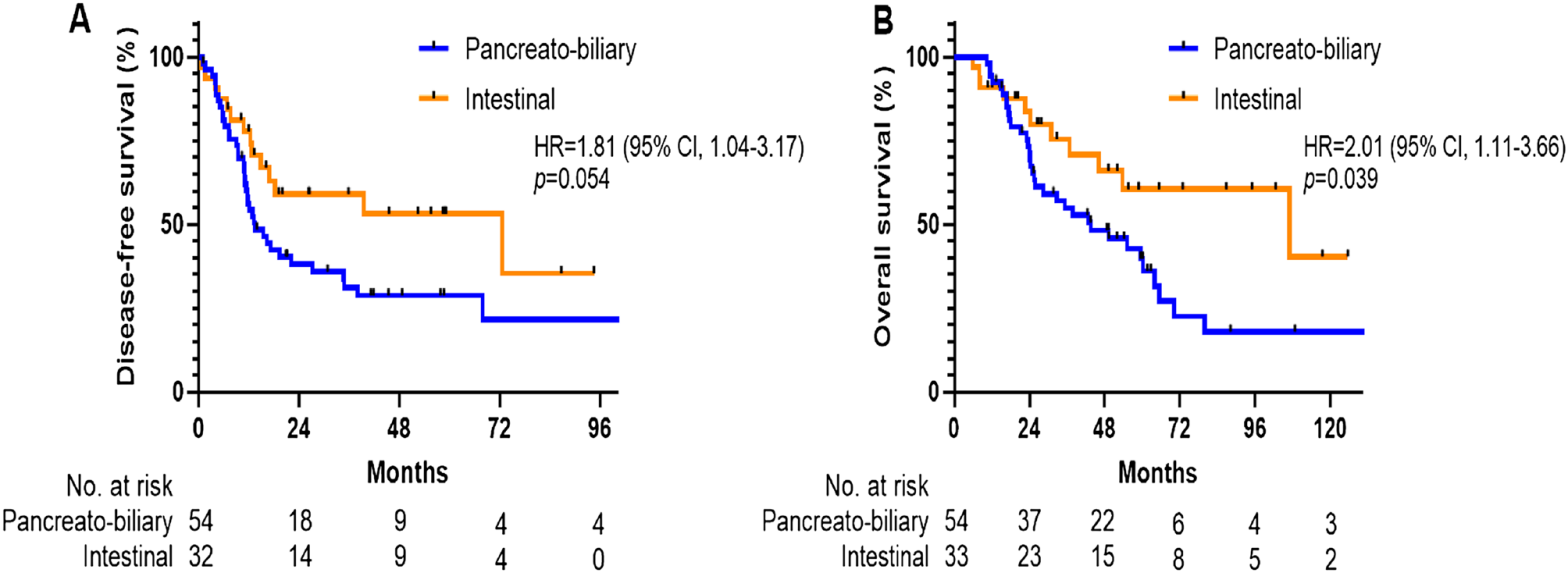
Kaplan–Meier estimates of disease-free survival (**A**) and overall survival (**B**) in patients with ampulla of Vater cancer, stratified by histologic subtypes.

**Figure 2:**
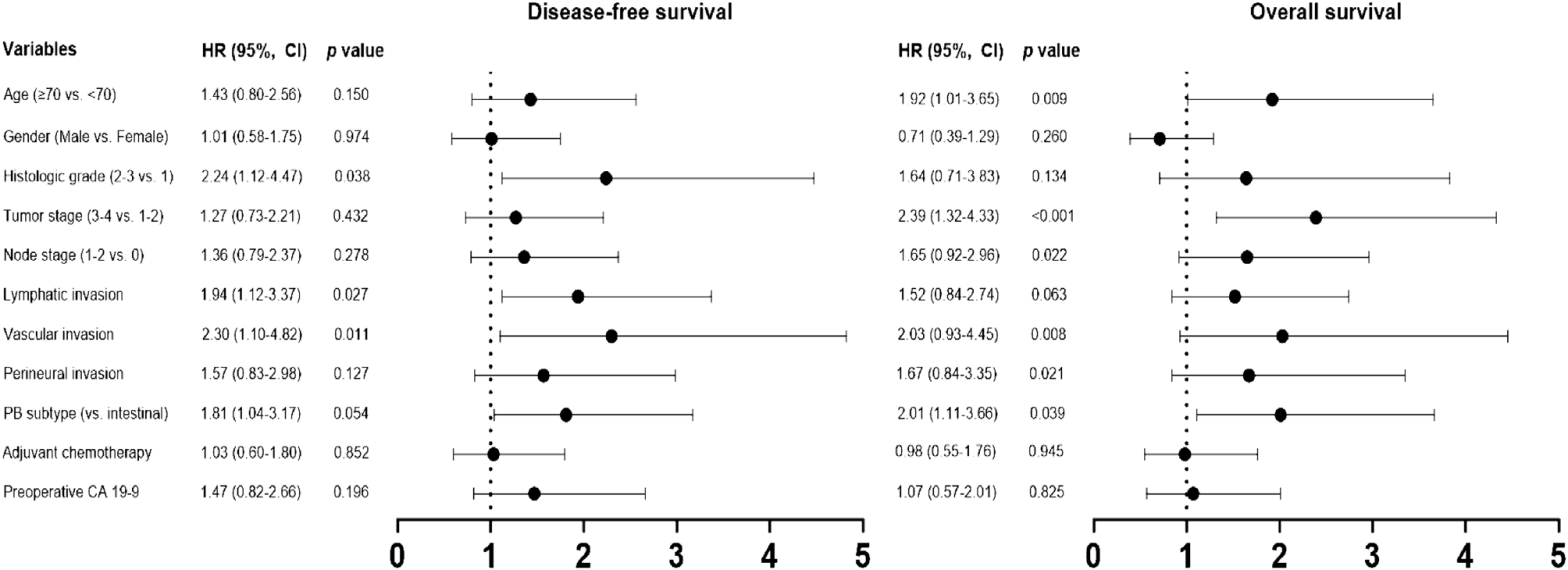
Forest plots for univariable analysis of disease-free survival and overall survival. Hazard ratios were estimated in a Cox proportional hazards regression model. HR, hazard ratio; PB, pancreato-biliary; CA 19–9, carbohydrate antigen 19–9.

In the survival analysis by histologic subtype, recurrence occurred in 37 (68.5%) of the 54 patients with the PB subtype and 15 (45.5%) of the 33 patients with the intestinal subtype. The median DFS was 13.4 months (95% CI, 8.10–18.7) in the PB group, compared to 72.7 months (95% CI, 0.53–144.8) in the intestinal group (HR = 1.81; 95% CI, 1.04–3.17; *p* = 0.054; Fig. 1A). Death events in the PB subtype and intestinal subtype were 36 (66.7%) of the 54 patients and 13 (39.4%) of the 33 patients, respectively. The median OS was 43.6 months (95%CI, 21.8–66.5) in the PB group, compared to 106.9 months (95% CI, 14.7–199.1) in the intestinal group (HR = 2.01; 95% CI, 1.11–3.66; *p* = 0.039; Fig. 1B). Additionally, Fig. 2 provides a forest plot delineating the univariate analysis results for DFS and OS across the respective subgroups. In the multivariable analysis, advanced tumor stage (HR = 1.91; 95% CI, 1.05–3.46; *p* = 0.034) and vascular invasion (HR = 1.95; 95% CI, 1.05–3.63; *p* = 0.036) were predictors of shorter DFS (Table 2). For OS, advanced tumor stage significantly impacted outcomes (HR = 2.17; 95% CI, 1.10–4.28; *p* = 0.026; Table 3). Although not statistically significant, patients with the PB subtype tended to exhibit inferior DFS (*p* = 0.222) and OS (*p* = 0.239) compared to those in the intestinal subtype.

**Table 2:** Univariate and multivariate assessments of the clinicopathologic features and histologic subtypes in relation to disease-free survival in patients with ampulla of Vater carcinoma.

Variables	DFS			
	Univariate analysis		Multivariate analysis	
	HR (95% CI)	*p* value	HR (95% CI)	*p* value
Age ≥ 70 (vs. < 70 year)	1.43 (0.80–2.56)	0.150		
Histologic grade 2–3 (vs. grade 1)	2.24 (1.12–4.47)	0.038	1.78 (0.67–4.72)	0.244
**Tumor stage 3 or 4 (vs. Stage 1 or 2)**	**1.27 (0.73**–**2.21)**	**0.432**	**1.91 (1.05**–**3.46)**	**0.034**
Nodal metastasis (vs. none)	1.36 (0.79–2.37)	0.278	1.10 (0.63–1.95)	0.736
Lymphatic invasion (vs. none)	1.94 (1.12–3.37)	0.027	1.12 (0.55–2.27)	0.760
**Vascular invasion (vs. none)**	**2.30 (1.10**–**4.82)**	**0.011**	**1.95 (1.05**–**3.63)**	**0.036**
Perineural invasion (vs. none)	1.57 (0.83–2.98)	0.127		
Pancreato-biliary type (vs. intestinal)	1.81 (1.04–3.17)	0.054	1.47 (0.79–2.72)	0.222
Received AC (vs. none)	1.03 (0.60–1.80)	0.852		

*DFS*, disease-free survival; *HR*, hazard ratio; *AC*, adjuvant chemotherapy.

Significant values are in [bold].

**Table 3:** Univariate and multivariate evaluations of clinicopathologic factors and histologic subtypes in determining overall survival for patients with ampulla of Vater carcinoma.

Variables	OS			
	Univariate analysis		Multivariate analysis	
	HR (95% CI)	*p* value	HR (95% CI)	*p* value
Age ≥ 70 (vs. < 70 year)	1.92 (1.01–3.65)	0.009	1.79 (0.95–3.35)	0.070
Histologic grade 2–3 (vs. grade 1)	1.64 (0.71–3.83)	0.134		
**Tumor stage 3 or 4 (vs. Stage 1 or 2)**	**2.39 (1.32**–**4.33)**	** < 0.001**	**2.17 (1.10**–**4.28)**	**0.026**
Nodal metastasis (vs. none)	1.65 (0.92–2.96)	0.022	1.20 (0.62–2.31)	0.594
Lymphatic invasion (vs. none)	1.52 (0.84–2.74)	0.063		
Vascular invasion (vs. none)	2.03 (0.93–4.45)	0.008	1.56 (0.78–3.11)	0.207
Perineural invasion (vs. none)	1.67 (0.84–3.35)	0.021	1.05 (0.51–2.13)	0.904
Pancreato-biliary type (vs. intestinal)	2.01 (1.11–3.66)	0.039	1.53 (0.75–3.10)	0.239
Received AC (vs. none)	0.98 (0.55–1.76)	0.945		

*OS*, overall survival; *HR*, hazard ratio; *AC*, adjuvant chemotherapy.

Significant values are in [bold].

### Patterns of recurrence

An examination of local and distant recurrence patterns based on histologic subtype was conducted, with the descriptive findings detailed in Table 4. Although not statistically significant, patients with the PB subtype exhibited a higher likelihood of experiencing only local recurrence (11 out of 37, 29.7%) compared to those with the intestinal subtype (1 out of 15, 6.7%). The relative risk for locoregional only recurrence for the PB subtype was 4.46-fold (95% CI, 0.63–31.6, *p* = 0.143). In cases of systemic disease, a significantly higher number of patients with elevated CA 19–9 levels were observed in the PB subtype compared to the intestinal subtype (*p* = 0.024), while no differences in CEA elevation were detected between the two groups. The sites and number of distant recurrences were comparable between both groups. The median OS from relapse was 15.4 months (95% CI, 8.10–21.3) in patients with only locoregional recurrence and 16.1 months (95% CI, 9.52–22.7) with distant recurrence, with no significant difference (HR = 0.92; 95% CI, 0.47–1.70; *p* = 0.810; Figure S1).

**Table 4:** Features of patients experiencing recurrence following surgical resection.

Variables	Total (n=52)	PB typ (n=37)	Intestinal type (n=15)	*p *value
Pattern of disease recurrence, n (%)				
Locoregional only	12 (23.1)	11 (29.7)	1 (6.7)	0.143
Distant	40 (76.9)	26 (70.3)	14 (93.3)	
Site of recurrence, n (%)				
Liver	28 (53.8)	20 (54.1)	8 (53.3)	0.962
Lung	17 (32.7)	12 (32.4)	5 (33.3)	0.950
Peritoneum	11 (21.2)	8 (21.6)	3 (20.0)	1.000
Distant lymph node	10 (19.2)	6 (16.2)	4 (26.7)	0.448
Number of metastasis sites, n (%)*				
One site	27 (67.5)	18 (69.2)	9 (64.3)	0.750
Two sites or more	13 (32.5)	8 (30.8)	5 (35.7)	
CA19-9 level at systemic disease, n (%)				
Within normal (<40U/mL)	24 (46.2)	13 (35.1)	11 (73.3)	**0.024**
Above normal (≥40U/mL)	23 (44.2)	20 (54.1)	3 (20.0)	
Missing data	5 (9.6)	4 (10.8)	1 (6.7)	
CEA level at systemic disease, n (%)				
Within normal (<3.8ng/mL)	28 (53.8)	19 (51.4)	9 (60.0)	0.537
Above normal (≥3.8ng/mL)	17 (32.7)	13 (35.1)	4 (26.7)	
Missing data	7 (13.5)	5 (13.5)	2 (13.3)	

*PB*, pancreato-biliary; CA 19–9, carbohydrate antigen 19–9; *CEA*, carcinoembryonic antigen. * In 40 patients with confirmed systemic recurrence.

Significant values are in [bold].

### Relapse treatment

Chemotherapy was administered at relapse in 38 of 52 patients (73.1%), 28 of 37 patients (75.7%) with PB subtype, and 10 of 15 patients (66.7%) with intestinal subtype. Among the 38 patients who received first-line palliative chemotherapy, 29 (76.3%) were treated with gemcitabine plus cisplatin, while 9 (23.7%) received capecitabine plus oxaliplatin regimen (Table 5). The objective response rate was 21.4% (6 of 28 patients) for the PB subtype and 50.0% (5 of 10 patients) for the intestinal subtype.

**Table 5:** Effectiveness of first-line systemic chemotherapy in patients with advanced ampulla of Vater carcinoma.

Variables	Total	PB type	Intestinal type	*p* value
	(*n* = 38)	(*n* = 28)	(*n* = 10)	
First relapse chemotherapy, n (%)				
Gemcitabine/Cisplatin	29 (76.3)	23 (82.1)	6 (60.0)	0.507
Capecitabine/Oxaliplatin	9 (23.7)	5 (17.9)	4 (40.0)	
Best response, n (%)				
Partial response	11 (28.9)	6 (21.4)	5 (50.0)	
Stable disease	20 (52.6)	16 (57.1)	4 (40.0)	
Progressive disease	7 (18.5)	6 (21.5)	1 (10.0)	
Objective response rate, n (%)	11 (28.9)	6 (21.4)	5 (50.0)	0.087
Disease control rate, *n* (%)	31 (81.5)	22 (78.5)	9 (90.0)	0.65
Median PFS, months [95% CI]	5.5 [4.0–7.0]	5.0 [3.8–6.1]	6.1 [2.7–9.4]	0.255
6-months PFS, % [95% CI]		39.3 [21.7–56.5]	70.0 [32.9–89.2]	
Median OS, months [95% CI]	14.3 [7.4–21.3]	10.3 [6.9–13.5]	28.3 [9.2–47.4]	**0.025**
12-months OS, % [95% CI]		42.9 [24.6–60.0]	90.0 [47.3–98.5]	

*PB*, pancreato-biliary; *PFS*, progression-free survival; *OS*, overall survival.

Significant values are in [bold].

**Figure 3:**
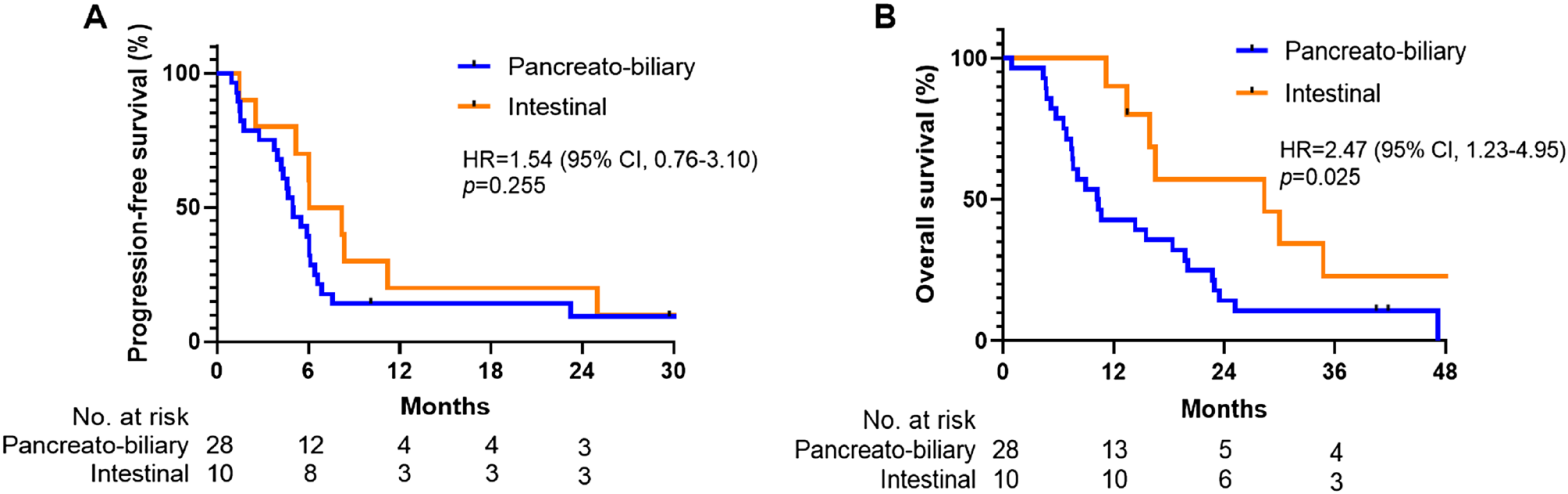
Kaplan–Meier estimates of progression-free survival and overall survival according to histologic subtype. (**A**) The median progression-free survival was 5.0 months in the pancreato-biliary subtype, as compared with 6.1 months in the intestinal subtype. (**B**) The median overall survival was 10.3 months in the pancreato-biliary group, as compared with 28.3 months in the intestinal group.

The analysis of progression-free survival (PFS) was based on 34 events among 38 patients (89.5%). The median PFS was 5.0 months (95% CI, 3.8–6.1) in the PB group as compared with 6.1 months (95% CI, 2.7–9.4) in the intestinal group (HR = 1.54; 95% CI, 0.76–3.10; *p* = 0.255; Fig. 3A). PFS rates at 6 and 12 months were 39.3% and 14.3% for the PB group, and 70.0% and 20.0% for the intestinal group, respectively. The median follow-up duration since the initiation of first-line systemic chemotherapy was 13.9 months (95% CI, 8.9–20.1). The OS analysis, starting from the administration of palliative chemotherapy, included 33 deaths among 38 patients (86.8%). The median OS was 10.3 months (95% CI, 6.9–13.5) in the PB group as compared with 28.3 months (95% CI, 9.2–47.4) in the intestinal group (HR = 2.47; 95% CI, 1.23–4.95; *p* = 0.025; Fig. 3B). OS rates at 6 and 12 months were 78.6% and 42.9% for the PB subtype, and 100.0% and 90.0% for the intestinal subtype, respectively. In patients who received gemcitabine plus cisplatin as their first-line systemic chemotherapy, patients with the PB subtype displayed poorer OS outcomes compared to those with the intestinal subtype (HR = 2.96; 95% CI, 1.32–6.62; *p* = 0.029 Figure S2, Table S1). However, no significant differences were observed in other efficacy parameters between the two groups.

## Discussion

In this investigation of surgically resected stage IB-III AoV cancer patients, it was observed that individuals with the PB subtype experienced poorer survival outcomes compared to those with the intestinal subtype. Regarding recurrence patterns, the majority of local recurrences were found in the PB subtype, while the intestinal subtype primarily exhibited systemic recurrence patterns. Furthermore, among patients who received palliative chemotherapy for systemic disease, those with the intestinal subtype demonstrated superior OS outcomes in comparison to the PB subtype. Notably, this is the first study to explore recurrence and survival outcomes in relation to histologic subtypes for a selected cohort of surgically resected AoV cancer patients at risk of recurrence, specifically excluding early stage IA patients.

In our cohort analysis, the PB subtype demonstrated a higher predominance relative to the intestinal subtype. These results are consistent with previous studies that differentiated subtypes utilizing IHC techniques within Western populations^9,11^. Likewise, investigations centered on East Asian subjects (Korean and Japanese) have reported similar findings^12^. Although biliary tract cancer, particularly extrahepatic cholangiocarcinoma, has the highest incidence in East Asia, the differences in histomolecular phenotype ratios for AoV cancer based on ethnic disparities remain unclear.

The prognostic implication of histological classification has been explored, yielding inconsistent outcomes^3–5,9^. Previously reported studies incorporated a significant number of early-stage patients exhibiting low recurrence likelihood, and a considerable proportion of patients were classified as mixed type, thereby complicating the precise assessment of the histological subtype's prognostic relevance. Research by Mafficini et al*.* revealed no association between histological subtype and prognosis, while specific molecular alterations emerged as negative survival predictors irrespective of the histological subtype^9^. In contrast, a recently published meta-analysis indicated that patients with the PB subtype were linked to advanced pathological stages and exhibited shorter OS relative to those with the intestinal subtype^12^. Our investigation has the advantage of evaluating the prognostic influence based on histologic subtype within a comparatively homogeneous cohort, as it excludes early-stage patients with low recurrence probability.

In the evaluation of histopathologic parameters, no statistically significant disparities were observed between the two cohorts, however in terms of tumor categories, a trend towards more advanced stages was evident in patients with the PB subtype. In a previous report, small-sized PB subtypes tended to invade more aggressively compared to small-sized intestinal subtypes^4^. Additionally, ampullary carcinomas with the PB subtype more often displayed infiltrative gross morphologic features as observed in the magnetic resonance imaging study^13^. The infiltrative tendency of the PB subtype could be connected to the findings that most local recurrences occur predominantly in the PB subtype rather than the intestinal subtype, even in cases where most patients have undergone R0 resection.

Adjuvant chemotherapy is considered for surgically resected AoV cancer patients with a risk of recurrence, such as advanced tumor stage, node metastasis, and R1 resection. Given the lack of consensus, fluorouracil-based or gemcitabine-based adjuvant chemotherapy is administered at the physician's discretion^14^. The randomized phase III ESPAC-3 trials identified a survival benefit with adjuvant gemcitabine-based chemotherapy, but the applicability of these results is limited due to the inclusion of patients with biliary tract and other periampullary cancers, as well as a significant number of patients with stage I or IVA cancer^15^. In certain centers, the selection of adjuvant chemotherapy may be driven by histological subtype. Previous studies have suggested that patients with PB or mixed subtype could potentially benefit from gemcitabine-based adjuvant chemotherapy. However, the interpretation of these retrospective studies, characterized by limited patient numbers and varied adjuvant regimens, should be approached with caution^16,17^.

Currently, only oral fluorouracil drugs such as capecitabine or S-1 (a combination of tegafur, gimeracil, and oteracil potassium) are recommended as standard adjuvant treatments for biliary tract cancer. In contrast, gemcitabine-based adjuvant treatment has not yielded clinical benefits in several trials^18^. Notably, in the recent phase III ASCOT trial, approximately 17% of the entire cohort were AoV cancer, and a significant survival advantage was observed in subgroup analysis for those receiving adjuvant S-1 treatment^19^. As a result, oral fluorouracil-based adjuvant chemotherapy might be a viable option even for patients with a PB subtype. However, given that oral fluorouracil-based adjuvant chemotherapy does not affect the reduction of local recurrence risk^20^, patients with the PB subtype of AoV cancer could consider adjuvant chemoradiotherapy or perioperative treatment to mitigate the risk of local recurrence.

In patients who received palliative chemotherapy for advanced disease, the PB subtype exhibited worse OS compared to the intestinal subtype. Nonetheless, the absence of a significant difference in systemic chemotherapy effectiveness between the two subgroups suggests that the favorable tumor biology of the intestinal subtype, rather than a histologic subtype-dependent response to systemic chemotherapy, plays a role even in advanced settings. Although the gemcitabine plus cisplatin regimen is anticipated to yield better results for the PB subtype relative to the intestinal subtype, no difference in chemotherapy effectiveness was observed between the groups when investigating only patients who used gemcitabine plus cisplatin as first-line treatment.

This study presents several limitations. Firstly, in the case of the mixed subtype, we categorized phenotypes according to the prevailing histologic features based on histology and IHC findings, but these might display clinical traits that diverge from each well-defined subtype. Secondly, we did not identify the predominant subtype of relapsed patients with initially ambiguous phenotypes, as no further biopsies were conducted after recurrence. Thirdly, the relationship between the node stage and survival outcomes was not distinctly evident in multivariate analysis. One potential reason could be the possible underestimation of the node stage, particularly in a subset of N0 stage patients who might not have undergone comprehensive nodal examinations. Lastly, the administration of adjuvant chemotherapy to certain patients at the physician's discretion could have impacted survival outcomes.

## Conclusions

Categorizing surgically treated AoV carcinoma patients based on histomolecular phenotypes seems to correlate with survival outcomes, patterns of recurrence, and prognoses even in advanced stages. Future prospective studies are essential to determine which patients with specific histomolecular subtypes could benefit from adjuvant therapies or intensified perioperative treatments.

## Methods

### Patients and data acquisition

The clinicopathological and outcome data of patients diagnosed with adenocarcinoma of the AoV who underwent curative pancreaticoduodenectomy with regional lymph node dissection at the Catholic University of Korea, Seoul St. Mary’s Hospital between August 2007 and December 2021 were analyzed. To be eligible for the study, patients had to meet the following criteria: (1) histologically confirmed adenocarcinoma of the AoV; (2) pathological stage IB-III according to the American Joint Committee of Cancer Staging, 8th edition^21^, and (3) confirmable recurrence and survival at the time of data collection. Patients with the following conditions were excluded: (1) pathological tumor, node, metastasis (TNM) stage IA disease; (2) no examined regional lymph node; (3) macroscopically remaining tumors after surgery (R2 resection); (4) received preoperative chemotherapy or radiotherapy before surgical resection; or (5) a secondary malignancy diagnosed after surgery.

### Histology

Formalin-fixed paraffin embedded (FFPE) tissue specimens from patients diagnosed with adenocarcinoma of the AoV were retrieved from the pathological archives. All pathologic features independently reviewed and confirmed by two specialists pancreatic histopathologists who were blinded to the clinical outcomes (SHL and YK). Tumors were classified into two histologic subtypes based on morphological features^22^. The intestinal subtype of adenocarcinoma was characterized by the presence of tall columnar cells forming elongated glands, which are similar in appearance to those seen in colorectal adenocarcinoma. The PB subtype was defined as having cells with rounded nuclei forming rounded glands, typical of pancreaticobiliary carcinomas. If a tumor displayed characteristics from both subtypes, it was classified as a mixed type.

### Tissue microarray analysis

During histopathological evaluation, areas that effectively represented the tumor were identified. From each FFPE tissue sample, at least two 2-mm diameter core tissues were obtained, preferably from different blocks. These core tissues were then methodically placed into a new recipient tissue microarray block using a trephine apparatus.

### Immunohistochemistry and evaluation

FFPE TMA sections underwent deparaffinization with xylene and were subsequently rehydrated via a series of descending alcohol concentrations. An automated Bond-max immunostainer (Leica Microsystems, Newcastle, UK) was utilized for immunostaining following antigen retrieval. The primary antibodies applied included anti-MUC1 monoclonal antibody (Novocastra, Newcastle, UK), anti-MUC2 monoclonal antibody (Novocastra, Newcastle, UK), anti-CDX2 monoclonal antibody (BioGenex, CA, USA), and anti-CK20 monoclonal antibody (Santa Cruz Biotechnology, TX, USA). Detection of antibody binding was performed using a Bond Polymer Refine Detection kit (catalog #DS9800; Leica Microsystems, Vista, CA, USA).

To define the AoV phenotype, four immunohistochemical markers were evaluated using IHC labeling on TMA samples, employing the following antibodies: MUC1, CDX2, CK20, and MUC2. Assessment included cytoplasmic immunoreactivity for CK20 and MUC2, cell surface staining for MUC1, and nuclear staining for CDX2. A modified H score (intensity × percentage of positive cells) greater than 35 indicated positive CDX2 expression, while the presence of any positive staining denoted positive MUC1, MUC2, and CK20 expression. Samples were initially categorized based on immunohistochemical staining outcomes. In cases where staining results were ambiguous, classification relied on aforementioned morphological evaluation of the whole slides^12^ and the criteria based on CDX2 and MUC1 expression^3^.

### Treatment

A pancreaticoduodenectomy or pylorus-preserving pancreaticoduodenectomy with standard lymph node dissection was performed based on the discretion of the surgeon. The decision to administer adjuvant chemotherapy after surgery and the choice of chemotherapy regimen were determined by the treating physician. Patients were administered fluorouracil-based adjuvant chemotherapy within 12 weeks post-surgery. Modifications to the chemotherapy dosage and schedule were permitted as deemed appropriate by the physician. Adverse events were evaluated in accordance with the National Cancer Institute's Common Terminology Criteria for Adverse Events, version 4.03.

### Surveillance

Postoperative patient assessments were conducted at 3-month intervals for the initial 2 years, biannually for the subsequent 3 years, and on an annual basis thereafter. Computed tomography imaging techniques were employed for imaging evaluations, and CA 19–9 and CEA levels were monitored during each visit. In cases where findings indicated potential recurrence, supplementary imaging or biopsies were performed to verify its presence.

### Statistical analysis

Descriptive statistics are reported as proportions or medians with range. Categorical variables were compared using the chi-square test or Fisher’s exact test, while continuous variables were compared using the unpaired t-test with Welch’s correction. DFS was defined as the interval between curative surgery and recurrence or death from any cause. OS was estimated from the date of surgery to the time of last follow-up or death from any cause. Survival outcomes were estimated using the Kaplan–Meier method and compared using the two-tailed log-rank test. Following univariate analysis, factors with a significant correlation to survival outcomes (*p* < 0.05), along with those previously established to have a clear association, were included in the multivariate analysis, employing the Cox proportional hazard regression model with adjustment for minimization factors. All the tests were two-sided, and *p* values < 0.05 were considered as statistically significant. Statistical analysis was performed using SPSS for Window version 24.0 (IBM SPSS Inc., Armonk, New York, USA) and GraphPad Prism version 8.0 (GraphPad Software Inc., San Diego, CA, USA).

### Ethics approval and consent to participate

The study conformed to the Korean regulations and the Declaration of Helsinki. Ethical approval for the acquisition of data was obtained the Institutional Review Board (IRB) of The Catholic University of Korea, Seoul St. Mary’s Hospital (approval ID: KC21RISI0518) with a waiver of informed consent due to the retrospective nature of the analysis.

### Supplementary Information

Supplementary Information.

## Data Availability

The datasets used in the current study are available from the corresponding author on request.
