# Supplementary material for: Histologic subtype-based evaluation of recurrence and survival outcomes in patients with adenocarcinoma of the ampulla of Vater
Source: Sci Rep. 2023 Oct 2;13:16547. doi: 10.1038/s41598-023-42386-6 (PMC10545688; doi:10.1038/s41598-023-42386-6)
Supplement: Supplementary file 1 — Supplementary Information. [file 41598_2023_42386_MOESM1_ESM.docx]

**Fig. S1 Kaplan-Meier estimates of overall survival in patients with ampulla of Vater cancer, stratified by recurrence patterns.**

**
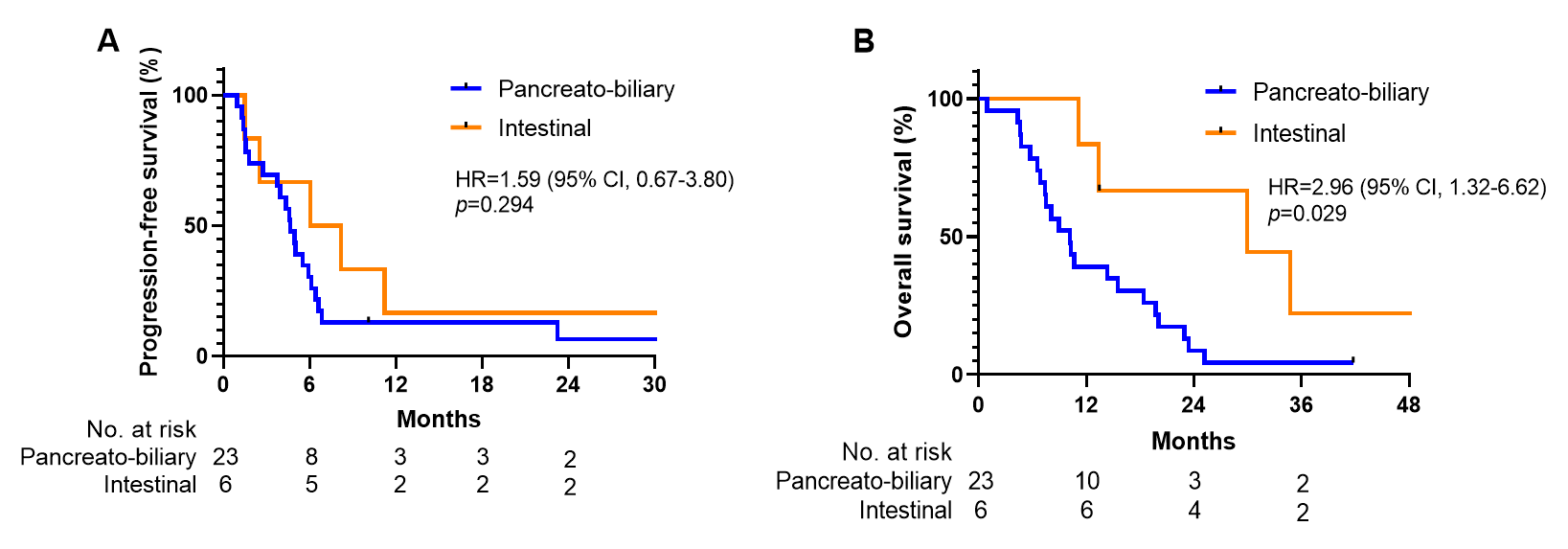
**

**Fig. S2 Kaplan-Meier estimates of progression-free survival and overall survival in patients with advanced ampulla of Vater cancer who received gemcitabine plus cisplatin as first-line systemic chemotherapy.** (A) The median progression-free survival was 4.7 months in the pancreato-biliary subtype, as compared with 7.1 months in the intestinal subtype. (B) The median overall survival was 10.2 months in the pancreato-biliary group, as compared with 30.0 months in the intestinal group.

**Table S1.** Efficacy of first-line treatment with cisplatin plus gemcitabine for patients with advanced ampulla of Vater carcinoma. PB, pancreato-biliary; PFS, progression-free survival; OS, overall survival.

| **Variables** | **Total**  **(n=29)** | **PB type**  **(n=23)** | **Intestinal type**  **(n=6)** | ***p* value** |
| --- | --- | --- | --- | --- |
| **Best response, n (%)**  Partial response  Stable disease  Progressive disease | 5 (17.2)  17 (58.6)  7 (24.2) | 3 (13.0)  14 (60.9)  6 (26.1) | 2 (33.3)  3 (50.0)  1 (16.7) |  |
| **Objective response rate, n (%)** | 5 (17.2) | 3 (13.0) | 2 (33.3) | 0.269 |
| **Disease control rate, n (%)** | 22 (75.9) | 17 (73.9) | 5 (83.3) | 1.000 |
| **Median PFS, months [95% CI]** | 5.0 [4.20-5.70] | 4.7 [3.73-5.58] | 7.1 [0.00-12.9] | 0.294 |
| **6-months PFS, % [95% CI]** |  | 30.4 [13.5-49.2] | 66.7 [19.5-90.5] |  |
| **Median OS, months [95% CI]** | 11.1 [5.67-16.6] | 10.2 [6.76-13.6] | 30.0 [0.00-62.4] | **0.029** |
| **12-months OS, % [95% CI]** |  | 39.1 [19.2-58.0] | 83.3 [27.3-97.4] |  |
